# Potentially inappropriate medication use and associated factors in residents of long-term care facilities: A nationwide cohort study

**DOI:** 10.3389/fphar.2022.1092533

**Published:** 2023-01-10

**Authors:** Suhyun Jang, Young-Mi Ah, Sunmee Jang, Yeji Kim, Ju-Yeun Lee, Jung-Ha Kim

**Affiliations:** ^1^ College of Pharmacy and Gachon Institute of Pharmaceutical Sciences, Gachon University, Incheon, South Korea; ^2^ College of Pharmacy, Yeungnam University, Gyeongsan, Gyeongbuk, South Korea; ^3^ Department of Statistics, Graduate School, Sungkyunkwan University, Seoul, South Korea; ^4^ College of Pharmacy and Research Institute of Pharmaceutical Sciences, Seoul National University, Seoul, South Korea; ^5^ Department of Family Medicine, Chung-Ang University Medical Center, Chung-Ang University College of Medicine, Seoul, South Korea

**Keywords:** potentially inappropriate medications, long-term care facilities, long-term care grade, determinants, comprehensive medication management, adverse drug reaction

## Abstract

**Background:** Residents in long-term care (LTC) facilities (LTCFs) may have multimorbidity and be unable to self-administer medication. Thus, due to the risk of potentially inappropriate medications (PIMs), epidemiological studies on PIM use and its associated factors should be conducted to ensure safe medication use for residents in LTCFs.

**Objective:** We evaluated PIM use among residents of LTCF and the associated factors in residents of LTCFs in Korea using a nationwide database.

**Methods:** This cross-sectional study used the Korea National Health Insurance Service Senior Cohort (KNHIS-SC) database 2.0 of the National Health Insurance Service (NHIS), a single public insurer in Korea. We analyzed older adults aged ≥65 years who were residents of LTCFs in 2018, using the KNHIS-SC database. The 2019 American Geriatrics Society (AGS) Beers criteria was used for PIM identification. The prevalence of PIM use was defined as the proportion of LTCF residents who received PIM prescriptions at least once. We evaluated the frequency of prescriptions, including PIMs, and determined the most frequently used PIMs. We also conducted a multivariable logistic regression analysis to identify the factors associated with PIM use.

**Results:** The prevalence of PIM among the LTCF residents was 81.6%. The prevalence of PIM was 74.9% for LTC grades 1 or 2 (high dependence) and 85.2% for LTC grades 3–5 (low dependence). Quetiapine was the most frequently prescribed PIM, followed by chlorpheniramine. The low dependence level was significantly associated with PIM use (odds ratio of LTC grades 3–5: 1.49, 95% confidence interval 1.32–1.68, reference: LTC grades 1 or 2); moreover, the number of medical institutions visited, and medications emerged as primary influencing factors.

**Conclusion:** Most LTCF residents were vulnerable to PIM exposure. Furthermore, exposure to PIMs is associated with LTC grade. This result highlights the need for comprehensive medication management of LTCF residents.

## 1 Introduction

The life expectancy of humans at birth has been increasing, and population aging is a global situation (The World Bank, 2020). In Korea, as of 2020, the life expectancy of newborns is 83 years and the proportion of the aged population (≥65 years) is 15.7%. Aging in Korea is progressing rapidly, and Korea is expected to become a super-aged society, with more than 20% of the population aged 65 years or older by 2025 (20.6% of the total population) (The World Bank, 2020; KOrean Statistical Information Service (KOSIS), 2022). Aged people are susceptible to adverse drug reactions (ADRs), and these ADRs could progress to severe disease; the pharmacokinetic and pharmacodynamic response to medications is altered in ADRs, and polypharmacy is common in older adults (Mangoni and Jackson, 2004; Wastesson et al., 2018). ADR-related emergency department visits in aged patients were twice that of the general population, and the risk of the severe ADR was approximately 7 times higher (Budnitz et al., 2006).

Older patients are vulnerable to medication harm, and some potentially inappropriate medications (PIMs) may carry a greater risk to patient than the benefits provided by those medications. Therefore, guidelines for older adults have been announced for PIMs. The Beers Criteria, STOPP/START, NORGEP, and PRISCUS are representative guidelines (Rognstad et al., 2009; Holt et al., 2010; O’Mahony et al., 2015; By the American Geriatrics Society Beers Criteria Update Expert, 2019). The use of PIMs in older adults is associated with ADRs, falls and fractures, hospitalizations, and increased healthcare costs (Hyttinen et al., 2016; Damoiseaux-Volman et al., 2021; Yadesa et al., 2021). Thus, medication management to evaluate and prevent PIM use in older adults is important. The prevalence of PIM use in older adults varies according to study population and guidelines. A systematic review of PIM use in older inpatients reported a prevalence of 30.4%–97.1%, and the prevalence of PIM use in community-dwelling older patients in the US was 42.6% (Davidoff et al., 2015; Redston et al., 2018). Residents in long-term care facilities (LTCFs) are thought to be the frailest members of society because they lose independence in daily life beyond a certain level and have a short life expectancy.

As the aging population increases, the number of residents in LTCF also increases. In Korea, the number of residents in LTCF has increased to 169,405 in 2018 after the adoption of social long-term care insurance (LTCI) in 2008. LTCI is mandatory social insurance and operates by the National Health Insurance Service (NHIS) in Korea. Older adults, as well as younger adults who require long-term care (LTC), are eligible for LTCI. Care needs were assessed by the NHIS based on the care need certification (CNC) system, which is a standardized 52-item functional assessment tool and procedure (Kim et al., 2013). LTCI assesses older adults for their LTC grade based on activities of daily living, cognition, behavioral problems, and need for nursing care and rehabilitation. The LTC grade was classified into six categories according to the degree of need for LTC services identified in the comprehensive evaluation results: Level 1 (older adults with complete dependence), Level 2 (severe dependence), Level 3 (moderate dependence), Level 4 (mild dependence), Level 5 (dementia patients with lighter physical dependence), and Level 6 (dementia patients using services for day-care centers or home-dwellings) (Kim and Kwon, 2021; National Health Insurance Service, 2021).

The prevalence of PIM use in LTCF was reported to be 18.5%–82.6% based on the Beers criteria (Storms et al., 2017) and the factors associated with PIM use in LTCF were age, duration of institutionalization, geriatric score, physician’s role, dementia, and polypharmacy (Anrys et al., 2018; Moreira et al., 2020). In Korea, 58.2% of residents of LTCFs used PIMs, and the number of co-medications and the LTCI grade were associated with PIM use, when data regarding 20 LTCFs were evaluated (Hwang et al., 2015). There is a need to develop strategies for safe medication use among residents in LTCFs. However, to the best of our knowledge, no study has comprehensively evaluated PIM use among LTCF residents in Korea. Considering that the impact of medication could be greater in frail older adults, PIM use should be evaluated according to the LTC level.

Therefore, we aimed to evaluate PIM use and its associated factors in residents of LTCFs in Korea using a nationwide database.

## 2 Methods

### 2.1 Database and populations

This cross-sectional study used data from the Korea National Health Insurance Service Senior Cohort (KNHIS-SC) database (DB) 2.0 of the National Health Insurance Service (NHIS), a single public insurer in Korea. This database included de-identified information for approximately 8% of the aged population (aged 60 years and older) from 2002 to 2019: sociodemographic data including age, sex, decile of insurance contribution of each subject and death event, healthcare utilization, such as hospitalization, outpatient visits, and medication prescription; results of national health screening services; and information on LTCI, such as LTC grade and type of service used.

This study included older adults who were admitted to LTCF for the first time in 2018 and defined the date of the first claim for LTCF use in 2018 for each patient as the index date. We used the LTCI grade as an indicator of the level of LTC needs. We excluded residents with LTC grade 6 (dementia patients using services for day-care centers or home-dwellings) because they could not use LTCF. We then classified them as LTCF residents based on their LTC grade: LTC grade 1 or 2 for patients with high dependence, and LTC grade 3–5 for patients with low dependence.

### 2.2 Variables

#### 2.2.1 Potentially inappropriate medications

We applied the criteria of “potentially inappropriate medication use in older adults” from the 2019 Beers criteria to identify PIM use in the study population; however, the PIM criteria for medications not approved in Korea were not considered (The 2019 American Geriatrics Society Beers Criteria^®^ Update Expert Panel, 2019). PIM use was identified by applying the guideline based on the diagnosis code or medication to evaluate PIM use accurately. The International Classification of Diseases 10th revision (ICD-10) codes are listed in Supplementary Table S1.

PIM use was identified for each outpatient prescription 1 year after the index date, excluding topical agents. The prevalence of PIM use in LTCF residents was calculated using the number of residents who were prescribed PIMs at least once a year and the total number of residents included as the numerator and denominator, respectively. In addition, we evaluated the frequency and duration of PIMs in each patient. The top ten ingredients were identified based on the number of prescriptions.

#### 2.2.2 Covariables

The characteristics of the included residents and LTCF were considered covariates in this study: 1) for residents, healthcare utilization, socio-demographic characteristics (age, sex, and insurance types), comorbidities using the Charlson Comorbidity Index (CCI) and other chronic diseases, number of medications prescribed, LTC grade, duration of LTCI beneficiaries (from the initial date of LTC grade evaluation to index date), and duration of residence in a LTCF (from the initial claim date of LTCF to index date); 2) for LTCF, the bed capacity, ownership type (private or other, local government, corporate), and number of contracted doctors of LTCF. Healthcare utilization, number of medications prescribed, CCI, and other chronic diseases were identified a year before the index date. Chronic diseases were defined as hospitalization (≥2 days) or two or more outpatient visits according to the main diagnosis code. The ICD-10 codes or ICD-10 of reference medications used are listed in Supplementary Table S1. Other variables, such as age, sex, insurance type, and LTC grade, were identified at the index date.

### 2.3 Analysis

The mean, standard deviation (SD), and percentage were used for descriptive statistics, and t-tests and chi-squared tests were used to identify differences between the groups. Multiple logistic regression was used to identify factors associated with PIM use among LTCF residents. SAS 9.4 (SAS Institute, Inc., Cary, NC, United States) was used for data management and statistical analyses. The statistical significance was analyzed at a *p*-value ≤ .05.

## 3 Results

**FIGURE 1:**
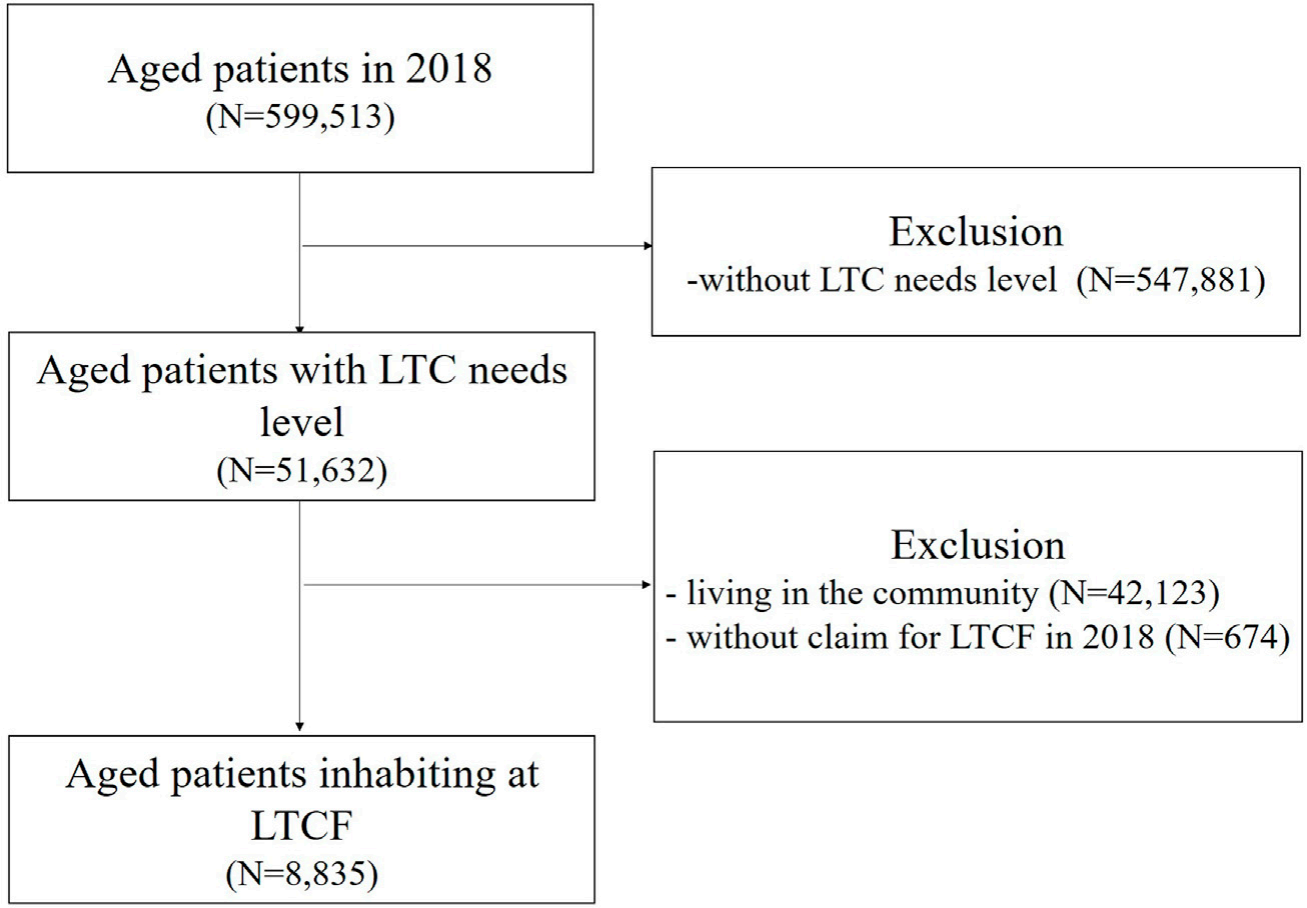
Flowchart for patient seletion. LTC, long-term care; LTCF, long-term care facility.

In 2018, 51,632 out of 599,513 older adults were LTCI beneficiaries, and 8,835 were LTCF residents (Figure 1). Women accounted for 77.8%, and the mean age was 82.1 years (SD 5.8). Approximately, half of the residents presented with dementia (57.6%) and hypertension (48.9%) as comorbidities, and 39.4% were hospitalized. The LTC grade of most residents was Level 3 (39.2%), the average LTCI beneficiary period was 52.1 months, and the average LTCF residence period was 36.4 months. Among the LTCF residents, 81.6% (n = 7,207) had at least one PIM prescription per year. The number of outpatient visits (23.4 vs. 14.0, *p* < .001), number of medical institutions used (3.0 vs. 1.9, *p* < .001), and number of medications (7.7 vs. 5.6, *p* < .001) were significantly higher for PIM users than for PIM non-users. A proportion of residents (32% and 47.5%) who were PIM users and non-users, respectively, had LTC grades 1–2 (with high dependence) (*p <* .001) (Table 1).

**TABLE 1:** Baseline characteristics of included long–term care facility residents in this study.

	Total (*N* = 8,835)		PIM user (*N* = 7,207)		No PIM user (*N* = 1,628)		*p*-value
	N	(%)	N	(%)	N	(%)	
Socio-demographics							
Sex, female	6,873	(77.8)	5,616	(77.9)	1257	(77.2)	.5319
Age (years)^a^	82.10 ± 5.8		82.02 ± 5.8		82.47 ± 5.7		.0048
65–74	986	(11.2)	830	(11.5)	156	(9.6)	.0417
75–84	4,300	(48.7)	3,514	(48.8)	786	(48.3)	—
85–89	3,031	(34.3)	2,456	(34.1)	575	(35.3)	—
≥90	518	(5.9)	407	(5.7)	111	(6.8)	—
Type of health insurance							
Medical insurance	7,017	(79.4)	5,658	(78.5)	1359	(83.5)	<.0001
Medical aid	1,818	(20.6)	1,549	(21.5)	269	(16.5)	—
CCI^a^	1.5 ± 1.1		1.5 ± 1.1		1.3 ± 1.1		<.0001
0	1,272	(14.4)	935	(13)	337	(20.7)	<.0001
1	4,046	(45.8)	3,332	(46.2)	714	(43.9)	—
2	2,271	(25.7)	1,876	(26)	395	(24.3)	—
3	848	(9.6)	723	(10)	125	(7.7)	—
4	272	(3.1)	229	(3.2)	43	(2.6)	—
≥5	126	(1.4)	112	(1.6)	14	(.9)	—
Chronic disease							
Dementia	5,091	(57.6)	4,370	(60.6)	721	(44.3)	<.0001
Mental disorders^b^	635	(7.2)	579	(8)	56	(3.4)	<.0001
Cerebrovascular disease	1,362	(15.4)	1,061	(14.7)	301	(18.5)	.0001
Cardiovascular disease	618	(7)	512	(7.1)	106	(6.5)	.3967
Parkinson’s disease	574	(6.5)	466	(6.5)	108	(6.6)	.8039
Hypertension	4,317	(48.9)	3,595	(49.9)	722	(44.4)	<.0001
Diabetes mellitus	1,834	(20.8)	1,561	(21.7)	273	(16.8)	<.0001
Dyslipidemia	943	(10.7)	761	(10.6)	182	(11.2)	.4642
Osteoarthritis	1,285	(14.5)	1,132	(15.7)	153	(9.4)	<.0001
Healthcare utilization							
Hospitalization	3,479	(39.4)	2,868	(39.8)	611	(37.5)	.0913
Number of outpatient visits^a^	21.7 ± 16.4		23.4 ± 16.7		14 ± 12.4		<.0001
Number of medical institutions visited^a^	2.8 ± 1.8		3 ± 1.9		1.9 ± 1.4		<.0001
≤2	4,709	(53.3)	1,187	(16.5)	3522	(216.3)	<.0001
3–4	2,799	(31.7)	339	(4.7)	2460	(151.1)	—
≥5	1,327	(15.0)	102	(1.4)	1225	(75.2)	—
Number of prescriptions^a^	21.5 ± 15.4		23.5 ± 15.6		12.5 ± 10.5		<.0001
Number of medications^a^	7.3 ± 3.7		7.7 ± 3.7		5.6 ± 3.4		<.0001
0–2	1,007	(11.4)	630	(8.7)	377	(23.2)	<.0001
3–4	1,392	(15.8)	1,053	(14.6)	339	(20.8)	—
5–9	4,486	(50.8)	3,741	(51.9)	745	(45.8)	—
≥10	1,950	(22.1)	1,783	(24.7)	167	(10.3)	—
Long-term care							
LTC grade							
1	1,010	(11.4)	682	(9.5)	328	(20.2)	<.0001
2	2,064	(23.4)	1,619	(22.5)	445	(27.3)	—
3	3,467	(39.2)	2,918	(40.5)	549	(33.7)	—
4	2,162	(24.5)	1,878	(26.1)	284	(17.4)	—
5	132	(1.5)	110	(1.5)	22	(1.4)	—
Duration of residence in LTCF (years)^a^	36.4 ± 30.7		35.6 ± 30.3		40.3 ± 31.8		<.0001
<1	1,982	(22.4)	1,657	(23.0)	325	(20)	<.0001
1≤, <2	2,049	(23.2)	1,724	(23.9)	325	(20)	—
2≤, <3	1,304	(14.8)	1,062	(14.7)	242	(14.9)	—
≥3	3,500	(39.6)	2,764	(38.4)	736	(45.2)	—
Duration of LTCI (years)^a^	52.1 ± 34.7		51.0 ± 34.5		57.2 ± 35.2		<.0001
<3	3,467	(39.2)	2,925	(40.6)	542	(33.3)	<.0001
3–5	2,182	(24.7)	1,796	(24.9)	386	(23.7)	—
≥5	3,186	(36.1)	2,486	(34.5)	700	(43)	—
Ownership type of LTCF							
Local government	419	(4.7)	331	(4.6)	88	(5.4)	.001
Corporate	3,797	(43)	3,041	(42.2)	756	(46.4)	—
Private, other	4,619	(52.3)	3,835	(53.2)	784	(48.2)	—
Number of contracted physicians							
0	3,764	(42.6)	3,085	(42.8)	679	(41.7)	.0679
1	3,545	(40.1)	2,907	(40.3)	638	(39.2)	—
2	1,182	(13.4)	951	(13.2)	231	(14.2)	—
≥3	344	(3.9)	264	(3.7)	80	(4.9)	—
Bed capacity^a^	43.1 ± 50.0		42.4 ± 49.0		46.4 ± 54.0		.0055
<20	3,734	(42.3)	3,047	(42.3)	687	(42.2)	.0003
20–49	2,187	(24.8)	1,834	(25.5)	353	(21.7)	—
50–99	2,015	(22.8)	1,623	(22.5)	392	(24.1)	—
100–199	709	(8)	565	(7.8)	144	(8.9)	—
≥200	190	(2.2)	138	(1.9)	52	(3.2)	—

^a^ Presented in mean ± standard deviation.

^b^ Mental disorders excluding dementia and Parkinson’s disease.

Abbreviations: CCI, charlson comorbidity index; LTC, long-term care; LTCF, long-term care facility; LTCI, long-term care insurance; PIM, potentially inappropriate medication.

Healthcare utilization and PIM use according to the LTC grade are shown in Table 2. Residents with LTC grades 3–5 had more outpatient visits, number of medical institutions visited, and prescriptions, compared to residents with LTC grade 1–2 (no. of outpatient visits 20.5 vs. 22.3, *p* < .001; no. of medical institutions visited 2.4 vs. 3.0, *p* < .001; and no. of outpatient visits 19.5 vs. 22.5, *p* < .001, respectively). Nevertheless, 74.9% and 85.2% of residents with LTC grade 1–2 and LTC grade 3–5 were PIM users, respectively (*p* < .001). The number of prescriptions with PIM and the prescription period for PIM use in LTC grade 3–5 resident PIM users were significantly higher than those in LTC grade 1–2 resident PIM users (no. of prescriptions 12.4 vs. 10.5, *p* < .001 and prescription period of PIM 250.5 vs. 215.8, *p* < .001, respectively).

**TABLE 2:** Healthcare utilization and potentially inappropriate medication use according to the LTC grade.

	Total (*N* = 8835)		LTC grade 1–2 (*N* = 3074)		LTC grade 3–5 (*N* = 5629)		*p*-value
	N	(%)	N	(%)	N	(%)	
Healthcare utilization							
Number of outpatient visits^a^	21.7 ± 16.4		20.5 ± 16.6		22.3 ± 16.3		<.0001
Number of medical institutions used^a^	2.8 ± 1.8		2.4 ± 1.6		3.0 ± 1.9		<.0001
≤2	4709	(53.3)	1897	(61.2)	2812	(48.8)	<.0001
3–4	2799	(31.6)	885	(28.8)	1914	(33.2)	—
≥5	1327	(15)	292	(9.5)	1035	(18)	—
Number of prescriptions^a^	21.5 ± 15.4		19.5 ± 15.4		22.5 ± 15.3		<.0001
Number of medications^a^	7.3 ± 3.7		7.0 ± 3.7		7.5 ± 3.8		<.0001
0–2	1007	(11.4)	411	(13.4)	596	(10.3)	<.0001
3–4	1392	(15.8)	537	(17.5)	855	(14.8)	—
5–9	4486	(50.8)	1527	(49.7)	2959	(51.4)	—
≥10	1950	(22.1)	599	(19.5)	1,351	(23.5)	—
Potentially inappropriate medications							
PIM user	7207	(81.6)	2301	(74.9)	4796	(85.2)	<.0001
Number of prescriptions with PIM^a^	11.8 ± 9.4		10.5 ± 8.8		12.4 ± 9.6		<.0001
1–2	1200	(16.7)	501	(21.8)	699	(12.1)	<.0001
3–5	961	(13.3)	337	(14.7)	624	(10.8)	—
6–10	1252	(17.4)	413	(18)	839	(14.6)	—
11–15	1958	(27.2)	562	(24.4)	1396	(24.2)	—
16–20	711	(9.9)	183	(8)	528	(9.2)	—
≥21	1125	(15.6)	305	(13.3)	820	(14.2)	—
Number of PIMs^a^	2.6 ± 1.6		2.3 ± 1.4		2.8 ± 1.7		<.0001
Number of days of PIM prescription^a^	239.4 ± 146.2		215.8 ± 150.6		250.5 ± 142.7		<.0001
≤30	1304	(18.1)	521	(22.6)	783	(13.6)	<.0001
31–180	1262	(17.5)	462	(20.1)	800	(13.9)	—
181–270	503	(7)	179	(7.8)	324	(5.6)	—
≥271	4138	(57.4)	1139	(49.5)	2999	(52.1)	—

^a^ Presented in mean ± standard deviation.

Abbreviation: PIM, potentially inappropriate medication.

The PIM prescribed most frequently to LTCF residents was quetiapine (22.8%), followed by chlorpheniramine (13.8%), and zolpidem (7.1%; Table 3). The top three PIM medications were the same regardless of LTC grade. No difference was found between the two groups in terms of the composition of the top ten PIMs within the different rankings.

**TABLE 3:** Top 10 potentially inappropriate medications according to the LTC grade.

No.	Total			LTC grade 1–2			LTC grade 3–5		
	(*N* = 127,266)^a^			(*N* = 34,829)^a^			(*N* = 92,437)^a^		
	Medications	No. of Rx	(%)	Medications	No. of Rx	(%)	Medications	No. of Rx	(%)
1	Quetiapine	29,052	(22.8)	Quetiapine	7384	(21.2)	Quetiapine	20,833	(23.1)
2	Chlorpheniramine	17,620	(13.8)	Chlorpheniramine	5969	(17.1)	Chlorpheniramine	9011	(10.0)
3	Zolpidem	9065	(7.1)	Zolpidem	2643	(7.6)	Zolpidem	6294	(7.0)
4	Risperidone	7028	(5.5)	Hydroxyzine	1758	(5.1)	Risperidone	5196	(5.8)
5	Diazepam	5281	(4.2)	Risperidone	1714	(4.9)	Diazepam	3798	(4.2)
6	Amitriptyline	5195	(4.1)	Diazepam	1429	(4.1)	Amitriptyline	3766	(4.2)
7	Hydroxyzine	4851	(3.8)	Clonazepam	1392	(4.0)	Glimepiride	3079	(3.4)
8	Clonazepam	4569	(3.6)	Amitriptyline	1357	(3.9)	Clonazepam	3078	(3.4)
9	Glimepiride	4092	(3.2)	Glimepiride	906	(2.6)	Hydroxyzine	3055	(3.4)
10	Dimenhydrinate	3345	(2.6)	Dimenhydrinate	687	(2.0)	Dimenhydrinate	2601	(2.9)

^a^ Total number of prescriptions including potentially inappropriate medications.

Abbreviation: Rx, prescriptions.

Table 4 shows the risk factors associated with the use of PIM. The older age group had a lower likelihood of PIM use than the group with individuals aged 65–74 years (Additional information of the model was reported in Supplementary Table S2). Medical aid beneficiaries and residents with mental disorders, including dementia, were associated with a higher likelihood of PIM use. LTC grade 3–5 was significantly associated with PIM use (odds ratio: 1.49, 95% CI: 1.32–1.68). As the number of medical institutions visited and medications increased, the likelihood of PIM prescriptions increased. The characteristics related to LTC, such as length of stay in LTCF, duration of LTCI, ownership type of LTCF, number of contracted physicians in LTCF, and bed capacity of LTCF, were not significantly affected by PIM use.

**TABLE 4:** Risk factors of potentially inappropriate medication use in long-term care facility residents.

		OR	95% CI	
Sex	Male	1	—	—
	Female	1.2	1.04	1.38
Age	65–74	1	—	—
	75–84	.79	.64	.96
	85–89	.78	.63	.96
	≥90	.71	.53	.96
Type of health insurance	Medical insurance	1	—	—
	Medical aid	1.22	1.05	1.43
CCI	0	1	—	—
	1	1.02	.85	1.23
	2	.89	.72	1.11
	≥3	.95	.73	1.24
Hospitalization	Yes	1	—	—
	No	1.1	.96	1.25
Number of medical institutions visited	≤2	1	—	—
	3–4	1.83	1.61	2.08
	≥5	3.11	2.56	3.78
Number of medications	0–2	1	—	—
	3–4	1.77	1.46	2.14
	5–9	2.92	2.47	3.45
	≥10	5.9	4.69	7.42
Chronic disease (References: None)	Dementia	2.05	1.77	2.36
	Mental disorders^a^	3.31	2.46	4.46
	Cerebrovascular disease	.81	.68	.97
	Cardiovascular disease	.78	.62	1
	Parkinson’s disease	.87	.68	1.11
	Hypertension	1.05	.94	1.19
	Diabetes mellitus	1.04	.89	1.22
	Dyslipidemia	.73	.61	.88
	Osteoarthritis	1.37	1.13	1.65
LTC grade	1–2	1	—	—
	3–5	1.49	1.32	1.68
Duration of residence in LTCF	<1	1	—	—
	1–2	1.05	.87	1.26
	2–3	1	.81	1.23
	≥3	1.13	.92	1.4
Duration of LTCI	<3	1	—	—
	3–5	0.9	.75	1.07
	≥5	.83	.69	1
Ownership type of LTCF	Local government	1	—	—
	Corporate	.92	0.7	1.2
	Private, other	1.01	.77	1.32
Number of contracted physicians	0	1	—	—
	1	.97	.82	1.14
	2	.97	.78	1.21
	≥3	.81	.59	1.12
Bed capacity	<20	1	—	—
	20–49	1.1	.92	1.33
	50–99	.92	.77	1.1
	≥100	.84	.66	1.07

^a^ Mental disorders excluding dementia and Parkinson’s disease.

Abbreviations: CCI, charlson comorbidity index; CI, confidence interval; LTC, long-term care; LTCF, long-term care facility; LTCI, long-term care insurance; OR, odds ratio.

## 4 Discussion

Most LTCF residents were vulnerable to PIM exposure. PIM users visited physicians more, visited various medical institutions, received more prescriptions, and took more medications than PIM non-users. Our results show that PIM use by LTCF residents in Korea (81.6%) was high. The high prevalence of PIM could be explained by differences in PIM criteria and population characteristics. The PIMs of the Beers criteria have been expanded as the criteria were revised (American Geriatrics Society Beers Criteria Update Expert Panel, 2012; American Geriatrics Society Beers Criteria Update Expert Panel et al., 2015). According to a systematic review (SR) reporting the range of PIM prevalence in LTCF, the PIM prevalence in the study using Beers 2012 criteria (58.2%–82.6%) was higher than that reported in studies using Beers pre-2012 criteria (18.5%–50.3%) (Storms et al., 2017). In addition, a recent study using Beers 2019 reported a 90.8% prevalence of PIM in nursing home residents (Díez et al., 2022). The prevalence of PIM use among older adults in Asian countries is relatively high. The prevalence of PIM was reported to be 66.7%–72.5% among older Chinese adults (Yang et al., 2015; Li et al., 2017), 77.2%–78.4% among older Japanese adults (Komagamine and Hagane, 2017; Komagamine et al., 2018), and 70.3%–81.0% among older Korean adults (Kim et al., 2016; Nam et al., 2016; Jang et al., 2021). One possible reason for the high prevalence of PIM was polypharmacy, which was associated with PIM according to the SR of PIM predictors (Tommelein et al., 2015). Asian countries have a cultural preference for medicines (Lo et al., 1994; Bates et al., 1995).

Furthermore, the risk of exposure to PIM is associated with the LTC grade, which indicates the degree of dependence in daily life. Residents with LTC grades 3–5 (low dependence) visited various medical centers and had more frequent outpatient visits and prescriptions than those with LTC grades 1 or 2 (high dependence). One possible explanation is that residents with low dependence could voluntarily use medical services more than those with high dependence. For LTCF residents in Korea, outpatient care can be received by a physician contracted with an LTCF; however, visiting an external medical institution, depending on the residents’ choices, is also possible. A previous study on LTCI beneficiaries in Korea reported similar results: those with low dependence (i.e., higher grades in social LTCI) had more outpatient visits, medications, and prescription days (Kang et al., 2021). The deprescribing effort for patients with limited life expectancy could also be an explanation. Deprescribing is an effort to reduce and manage inappropriate polypharmacy in older patients, which is more emphasized in frail older patients (Thompson and Farrell, 2013). According to a study that reported the cause of deprescribing according to physician specialties, the first reason for deprescribing by geriatricians was the limited benefit given the limited lifespan. This was different from the most common cause of deprescribing in other specialties such as ADRs (Goyal et al., 2020). Also, most patients and caregivers (77.6% and 76.4%, respectively) in Korea wanted to reduce the number of medications (Lee et al., 2022).

The factors associated with PIM use were sex; age; the beneficiaries of Medical Aid; number of medical institutions used; number of medications (or polypharmacy); and presence of dementia, mental disorders, or osteoarthritis, in line with previous studies (Vieira de Lima et al., 2013; Chang et al., 2014; Almeida et al., 2019; Moreira et al., 2020; Roux et al., 2020). Furthermore, we confirmed that residents with low dependence had a higher likelihood of PIM use. Hwang et al. showed that patients with low dependence (i.e., higher LTCI grade) had a higher likelihood of PIM use among older adults living in LTCF; however, this study was conducted for only 20 LTCFs (Hwang et al., 2015). Among overall senior patients, frail senior citizens (such as LTCF residents) are more likely to receive PIM prescriptions than normal senior citizens (Arnoldo et al., 2016; Maclagan et al., 2017); however, our results indicate that PIM use could be low in the frailer senior citizens among LTCF residents. Contrary to the private LTCFs, which exhibited a pattern of more PIM use than public LTCFs in a previous study (Liew et al., 2019), the characteristics of LTCF, such as ownership type, bed capacity, and the number of contracted physicians, were not associated with PIM use. As most LTCFs in Korea are for-profit facilities operated by corporations and private companies (95.3%), the insignificant effect of LTCF setting could be partially explained.

This study has several limitations. First, owing to the characteristics of the claims data, actual medication use could not be confirmed. However, given that the study participants inhabited LTCF, the difference between prescription information and actual medication intake may be minimal. Second, due to the lack of clinical data and absence of medical charts, the reasons for prescribing were not available. Therefore, we could not determine whether the physician decided that the benefits outweighed the risks of PIM use. Third, because the longitudinal cohort (KNHIS-SC DB) was constructed with patients in 2008, the included patients might have been older than the general aged population in Korea as LTCF residents in 2018. Considering the change in criteria for the LTC needs level in 2018 and the latest data on medication use, 2018 was selected as the study year, instead of the year when the cohort was constructed (2008).

## 5 Conclusion

The effective and safe use of medicine, particularly for frail older adults, such as LTCF residents, is crucial. We found most LTCF residents to be vulnerable to PIM exposure. Furthermore, exposure to PIMs is associated with LTC needs (i.e., LTC grade). Among the residents of LTCF, those with low LTC needs had more outpatient visits, medications, and PIM use. We should consider LTC and medical needs to optimize medications for senior LTCF residents. This result highlights the need for comprehensive medication management in LTCF residents.

## Data Availability

The data analyzed in this study is subject to the following licenses/restrictions: This study used data from the Korea National Health Insurance Service—Senior Cohort (KNHIS–SC) database 2.0 (NHIS–2022–2–073). Third-party data were obtained from the Korean National Health Insurance Service (KNHIS). The authors had no special access privileges to the data. Interested, qualified researchers can apply for access to the data by contacting the KNHIS. Requests to access these datasets should be directed to https://nhiss.nhis.or.kr/bd/ab/bdaba001cv.do.

## Abbreviations

ADR, Adverse drug reaction; CCI, Charlson Comorbidity Index; ICD-10, The International Classification of Diseases 10th revision; KNHIS-SC DB, Korea National Health Insurance Service Senior Cohort database; LTCF, Long-term care facility; LTCI, Long-term care insurance; NHIS, National Health Insurance Service; NSAID, non-steroidal anti-inflammatory inhibitor; OR, odds ratio; PIM, Potentially inappropriate medication; SD, standard deviation.
