# Supplementary material for: Potentially inappropriate medication use and associated factors in residents of long-term care facilities: A nationwide cohort study
Source: Front Pharmacol. 2023 Jan 10;13:1092533. doi: 10.3389/fphar.2022.1092533 (PMC9871308; doi:10.3389/fphar.2022.1092533)
Supplement: Supplementary file 1 [file DataSheet1.docx]

Supplementary Material

Potentially inappropriate medication use and associated factors in residents of long-term care facilities: A nationwide cohort study

Suhyun Jang^†^, Youngmi Ah^†^, Sunmee Jang*, Yeji Kim, Ju-Yeun Lee, Jung-Ha Kim

*** Correspondence:** Sunmee Jang: smjang@gachon.ac.kr

# Supplementary Tables

**Supplementary Table 1.** ICD-10 codes or medications used for disease definition.

| **Category of disease** | **ICD-10 codes or medication criteria** |
| --- | --- |
| Hypertension | I10~I13, I15 |
| Atrial fibrillation | I48 |
| Heart failure | I11.0, I13.0, I13.2, I50.x |
| Mental disorder | F code |
| Dementia | memantine, rivastigmine, galantamine, donepezil |
| Cerebrovascular disease | I60-I69 |
| Diabetes mellitus | E10-E14 |
| Parkinson disease | G20-G23 |
| Cardiovascular disease | I05-I09, I20-I27, I30-I52 |
| Dyslipidemia | E78 |
| Osteoarthritis | M00-M19, M45 |

ICD-10, The International Classification of Diseases 10th revision

**Supplementary Table 2.** Characteristics of logistic regression model.

|  |  | **β** | **SE β** | **Wald's χ2** | ***df*** | ***p*** | **OR** | **95% CI** | |
| --- | --- | --- | --- | --- | --- | --- | --- | --- | --- |
| **Constant** |  | -0.9406 | 0.2574 | 13.3497 | 1 | 0.0003 |  |  |  |
| **Sex** | Male |  |  |  |  |  | 1 |  |  |
|  | Female | 0.1722 | 0.0734 | 5.5077 | 1 | 0.0189 | 1.2 | 1.04 | 1.38 |
| **Age** | 65–74 |  |  |  |  |  | 1 |  |  |
|  | 75–84 | -0.2479 | 0.1045 | 5.6258 | 1 | 0.0177 | 0.79 | 0.64 | 0.96 |
|  | 85–89 | -0.2625 | 0.11 | 5.6933 | 1 | 0.017 | 0.78 | 0.63 | 0.96 |
|  | ≥90 | -0.3541 | 0.1515 | 5.462 | 1 | 0.0194 | 0.71 | 0.53 | 0.96 |
| **Type of health insurance** | Medical insurance |  |  |  |  |  | 1 |  |  |
|  | Medical aid | 0.1872 | 0.0788 | 5.6495 | 1 | 0.0175 | 1.22 | 1.05 | 1.43 |
| **CCI** | 0 |  |  |  |  |  | 1 |  |  |
|  | 1 | 0.0204 | 0.0953 | 0.0457 | 1 | 0.8307 | 1.02 | 0.85 | 1.23 |
|  | 2 | -0.1102 | 0.1123 | 0.9631 | 1 | 0.3264 | 0.89 | 0.72 | 1.11 |
|  | ≥3 | -0.0464 | 0.134 | 0.1198 | 1 | 0.7292 | 0.95 | 0.73 | 1.24 |
| **Hospitalization** | Yes |  |  |  |  |  | 1 |  |  |
|  | No | 0.0879 | 0.0658 | 1.7842 | 1 | 0.1816 | 1.1 | 0.96 | 1.25 |
| **Number of medical institutions visited** | ≤2 |  |  |  |  |  | 1 |  |  |
|  | 3–4 | 0.6033 | 0.0657 | 84.4176 | 1 | <.0001 | 1.83 | 1.61 | 2.08 |
|  | ≥5 | 1.1349 | 0.099 | 131.459 | 1 | <.0001 | 3.11 | 2.56 | 3.78 |
| **Number of medications** | 0–2 |  |  |  |  |  | 1 |  |  |
|  | 3–4 | 0.5708 | 0.0967 | 34.8503 | 1 | <.0001 | 1.77 | 1.46 | 2.14 |
|  | 5–9 | 1.0706 | 0.0855 | 156.688 | 1 | <.0001 | 2.92 | 2.47 | 3.45 |
|  | ≥10 | 1.7745 | 0.1168 | 230.756 | 1 | <.0001 | 5.9 | 4.69 | 7.42 |
| **Chronic disease** | Dementia | 0.7152 | 0.0737 | 94.062 | 1 | <.0001 | 2.05 | 1.77 | 2.36 |
| (Reference: None) | Mental disorders^a^ | 1.1979 | 0.1512 | 62.753 | 1 | <.0001 | 3.31 | 2.46 | 4.46 |
|  | Cerebrovascular disease | -0.2069 | 0.0882 | 5.4989 | 1 | 0.019 | 0.81 | 0.68 | 0.97 |
|  | Cardiovascular disease | -0.243 | 0.1214 | 4.0061 | 1 | 0.0453 | 0.78 | 0.62 | 1 |
|  | Parkinson’s disease | -0.1377 | 0.1237 | 1.238 | 1 | 0.2659 | 0.87 | 0.68 | 1.11 |
|  | Hypertension | 0.0516 | 0.0607 | 0.7225 | 1 | 0.3953 | 1.05 | 0.94 | 1.19 |
|  | Diabetes mellitus | 0.0425 | 0.0814 | 0.2726 | 1 | 0.6016 | 1.04 | 0.89 | 1.22 |
|  | Dyslipidemia | -0.3176 | 0.0947 | 11.2392 | 1 | 0.0008 | 0.73 | 0.61 | 0.88 |
|  | Osteoarthritis | 0.3117 | 0.0974 | 10.2538 | 1 | 0.0014 | 1.37 | 1.13 | 1.65 |
| **LTC grade** | 1–2 |  |  |  |  |  | 1 |  |  |
|  | 3–5 | 0.4002 | 0.0612 | 42.7521 | 1 | <.0001 | 1.49 | 1.32 | 1.68 |
| **Duration of residence in LTCF** | <1 |  |  |  |  |  | 1 |  |  |
|  | 1–2 | 0.0499 | 0.0942 | 0.2805 | 1 | 0.5964 | 1.05 | 0.87 | 1.26 |
|  | 2–3 | -0.0051 | 0.1073 | 0.0023 | 1 | 0.9618 | 1 | 0.81 | 1.23 |
|  | ≥3 | 0.1248 | 0.1075 | 1.3491 | 1 | 0.2454 | 1.13 | 0.92 | 1.4 |
| **Duration of LTCI** | <3 |  |  |  |  |  | 1 |  |  |
|  | 3–5 | -0.1089 | 0.0902 | 1.4571 | 1 | 0.2274 | 0.9 | 0.75 | 1.07 |
|  | ≥5 | -0.1832 | 0.0953 | 3.6955 | 1 | 0.0546 | 0.83 | 0.69 | 1 |
| **Ownership type of LTCF** | Local government |  |  |  |  |  | 1 |  |  |
|  | Corporate | -0.0874 | 0.1359 | 0.4142 | 1 | 0.5198 | 0.92 | 0.7 | 1.2 |
|  | Private, other | 0.0096 | 0.1381 | 0.0048 | 1 | 0.9447 | 1.01 | 0.77 | 1.32 |
| **Number of contracted physicians** | 0 |  |  |  |  |  | 1 |  |  |
|  | 1 | -0.0337 | 0.0845 | 0.159 | 1 | 0.69 | 0.97 | 0.82 | 1.14 |
|  | 2 | -0.0294 | 0.1109 | 0.0704 | 1 | 0.7907 | 0.97 | 0.78 | 1.21 |
|  | ≥3 | -0.2095 | 0.1642 | 1.6273 | 1 | 0.2021 | 0.81 | 0.59 | 1.12 |
| **Bed capacity** | <20 |  |  |  |  |  | 1 |  |  |
|  | 20–49 | 0.0968 | 0.0950 | 1.0381 | 1 | 0.3083 | 1.1 | 0.92 | 1.33 |
|  | 50–99 | -0.0849 | 0.0934 | 0.8255 | 1 | 0.3636 | 0.92 | 0.77 | 1.1 |
|  | ≥100 | -0.1734 | 0.1216 | 2.034 | 1 | 0.1538 | 0.84 | 0.66 | 1.07 |
| **Test** |  |  |  | χ2 | *df* | *p* |  |  |  |
| Overall model evaluation | |  |  |  |  |  |  |  |  |
| Likelihood ratio test | |  |  | 979.929 | 37 | <.0001 |  |  |  |
| Score test | |  |  | 950.944 | 37 | <.0001 |  |  |  |
| Wald test | |  |  | 815.235 | 37 | <.0001 |  |  |  |
| **Model Fit Statistics** | |  |  | Intercept Only | Intercept and Covariates | | |  |  |
| AIC |  |  |  | 8444.77 | 7538.843 | | |  |  |
| SC |  |  |  | 8451.85 | 7808.129 | | |  |  |
| -2 Log L |  |  |  | 8442.77 | 7462.843 | | |  |  |
